# Dynamics of Nanomotors Propelled by Enzyme Cascade Reactions

**DOI:** 10.3390/ijms252312586

**Published:** 2024-11-22

**Authors:** Jia-Qi Hu, Rui Zhao, Ru-Fei Cui, Jian-Long Kou, Jiang-Xing Chen

**Affiliations:** 1 School of Physics, Hangzhou Normal University, Hangzhou 311121, China; 2022111030010@stu.hznu.edu.cn (J.-Q.H.); 2023111030014@stu.hznu.edu.cn (R.Z.); 2 Department of Physics, Hangzhou Dianzi University, Hangzhou 310027, China; cuirufei@hdu.edu.cn; 3 Institute of Condensed Matter Physics, Zhejiang Institute of Photoelectronics and Zhejiang Institute for Advanced Light Source, Zhejiang Normal University, Jinhua 321004, China; kjl@zjnu.cn

**Keywords:** enzyme cascade reactions, nanomotor, multiparticle collision dynamics, self-propulsion, biosafety

## Abstract

Enzyme-powered nanomotors have attracted significant attention in materials science and biomedicine for their biocompatibility, versatility, and the use of biofuels in biological environments. Here, we employ a hybrid mesoscale method combining molecular dynamics and multi-particle collision dynamics (MD–MPC) to study the dynamics of nanomotors powered by enzyme reactions. Two cascade enzymes are constructed to be layered on the same surface of a Janus colloid, providing a confined space that greatly enhances reaction efficiency. Simulations indicate that such a configuration significantly improves the utilization of intermediate products and, consequently, increases the self-propulsion of the Janus motor. By presenting the gradient fields of substrates and products, as well as the hydrodynamics surrounding the motor, we explore the underlying mechanism behind the enhanced autonomous velocity. Additionally, we discuss the improvements in environmental safety of the modified motor, which may shed light on the fabrication of biocatalytic nano-machines in experiments.

## 1. Introduction

Enzyme-powered micro- and nanomotors harness reactions to catalyze various substrate fuels (such as glucose, hydrogen peroxide, urea) and convert chemical energy into mechanical energy, thereby achieving self-propulsion and directional movement [1,2,3,4,5,6,7,8]. Compared to traditional inorganic micro- and nanomotors, enzyme-powered versions not only offer the advantage of autonomous, rapid directional motion but also exhibit biocompatibility, biodegradability, and lower toxicity. These characteristics make such small motors promising for a wide range of applications, including the synthesis of nanomaterials, microelectronics fabrication, and biosensing [9,10,11,12,13,14].

Ma et al. studied single enzyme-powered micro/nanomotors [15]. Patiño et al. investigated the dynamics of non-Janus particles with an asymmetric enzyme distribution on the motor surface [16]. Various other configurations of enzyme motors, including tubular, rod-like, shell-like, flask-like, and stomatocytes, have also been studied recently [17,18,19,20,21]. Recent developing research has shown that the use of multiple enzyme-powered systems provides multiple driving forces, which is expected as a promising approach to enhancing propulsion and environmental safety. Various enzymes can independently catalyze corresponding substrates simultaneously, providing multiple sources of propulsion and enabling diverse functions. For example, glucose oxidase ( $GO_{x}$ ) and trypsin can utilize glucose and peptides, respectively, as their biofuels to jointly drive the movement of Janus swimmers [22]. Many other similar examples have been successfully implemented in previous experiments [23,24,25,26]. Unlike the dependence of multi-enzyme systems on various substrate sources, enzyme cascade reactions (ECRs) require only a single substrate source to propel motor motion in a multi-step manner [27,28,29,30]. For instance, by co-modifying the motor surface with $GO_{x}$ and catalase ( Cat ), $GO_{x}$ catalyzes the decomposition of glucose to drive the motor, while the intermediate product hydrogen peroxide ( $H_{2}O_{2}$ ) serves as a substrate fuel for Cat , further providing driving energy [27,31,32]. Using this ECRs, Lin et al. presented a generally efficient approach to facilitating the targeted delivery of nanoagents for the effective management of diabetic wounds by utilizing wound heterogeneity [33]. Recent studies have also explored other ECRs, such as Cat and horseradish peroxidase ( HRP ), $GO_{x}$ and bilirubin oxidase ( BOD ), and HRP and cytochrome c ( Cyt c ) [29,34,35]. This type of motor enhances the operability and safety of colloidal motors in biomedical applications.

In this study, we propose a new configuration of self-propelled motors by layering additional enzymes on the surface of Janus spheres to form ECRs. We investigate the effects of ECRs on the self-propulsion of the motor. Furthermore, the influence of substrate and intermediate product concentration distributions is analyzed, along with the fluid flow surrounding the motor. The distinctive characteristics of this configuration are presented, and the autonomous swimming behavior by self-diffusiophoresis is explored.

## 2. Results and Discussion

**Figure 1:**
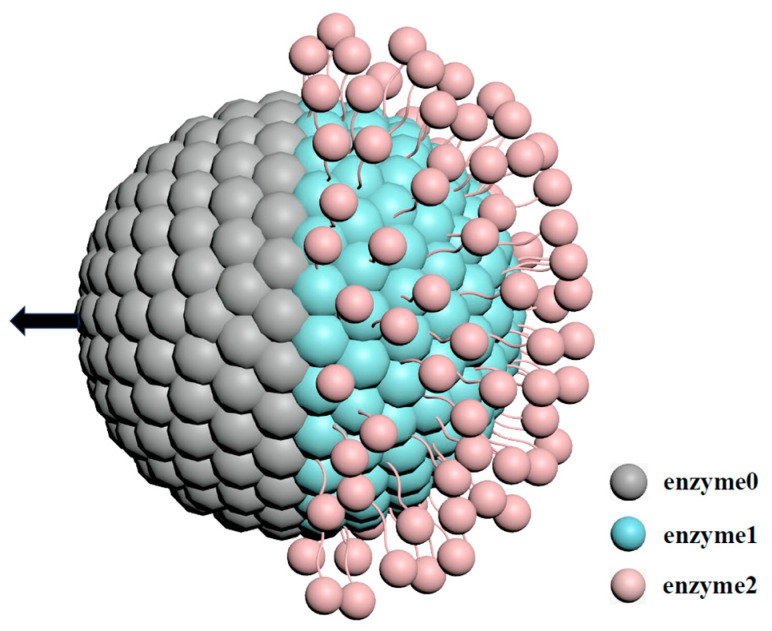
Schematic representation of the ECR motor model. The motor comprises an internal core structure and an outer shell populated with enzyme beads. These enzymes are uniformly distributed on the surface of a spherical shell with a radius of $R_{1}=2.9$ . Inert enzyme beads, denoted as $E_{0}$ (gray), have a radius of $\sigma_{1}=0.3$ and a total number of $N_{E_{0}}=500$ . Catalytic enzyme beads, designated as $E_{1}$ (green), possess the same radius and quantity ( $N_{E_{1}}=500$ ). A secondary catalytic enzyme type, $E_{2}$ (pink), with a radius of $\sigma_{2}=0.2$ , is arranged on the surface of an additional spherical layer with a radius of $R_{2}=4.2$ .

Our investigations of motor dynamics propelled by ECRs use a coarse-grain microscopic description of the entire system. We consider a colloid with two types of enzymes coated on its surface. One of the enzymes $E_{0}$ is inert, while the other, enzyme $E_{1}$ (mimicking $GO_{x}$ ), catalyzes the fuel A (mimicking glucose) into an intermediate product I (mimicking $H_{2}O_{2}$ ). Such Janus colloid has been fabricated in recent experiments. In addition, we construct a special structure by grafting another enzyme, $E_{2}$ (mimicking Cat ), onto the outer layer of $E_{1}$ , which catalyzes the intermediate product I into the final product B (mimicking $O_{2}$ ). A schematic diagram of the motor configuration is presented in Figure 1. The nanomotor is operated by a diffusiophoretic mechanism where the ECRs on the active enzymes produce concentration gradients that drive propulsion. The solution in which the motor moves contains fuel A as well as products I and B particles. These species interact with the motor through intermolecular potentials and determine how the motors move in the presence of chemical gradients produced by the motors themselves. Additional information concerning the motor configuration and the simulation is given in the Section 3.

**Figure 2:**
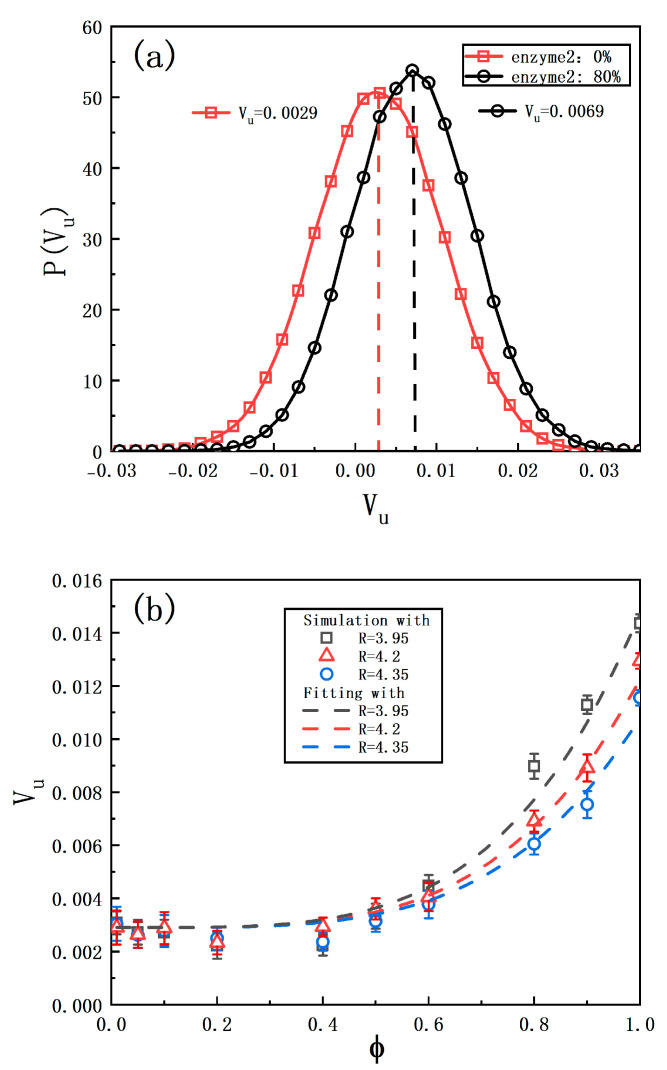
(**a**) Probability distribution of $V_{u}$ at $R_{2}=4.2$ with ϕ=0 (squares) and ϕ=0.8 (circles). (**b**) Average $V_{u}$ of the motor as a function of ϕ for different $R_{2}$ . The parameter ϕ is defined by $\frac{N_{E_{2}}{\left(2^{1/6}\sigma_{2}\right)}^{2}}{2R_{2}^{2}}$ as the area percentage of $E_{2}$ uniformly distributed on the second layer. The data are plotted from 20 independent realizations.

When fuel particles A diffuse into the reaction zone of enzyme $E_{1}$ , they are converted into intermediate particles I under the catalytic effect, creating an asymmetric distribution of A and I particles around the Janus colloid, which account for the self-propulsion due to the inequality of the interaction energies ( $\epsilon_{A\beta }<\epsilon_{B\beta }$ ). We define the unit vector $\hat{u}$ from the center of mass of the $E_{1}$ hemisphere to that of the $E_{0}$ hemisphere, as illustrated by the arrow in Figure 1. The average velocity $V_{u}$ along this axis can be calculated by $V_{u}=V\left(t\right)\cdot \hat{u}$ , where $V\left(t\right)$ represents the instantaneous velocity of the colloid’s center of mass, and the brackets denote a time average. Figure 2a shows the probability distribution of $V_{u}$ , indicating that the velocity along $\hat{u}$ is approximately $V_{u0}=0.0029$ . In previous experiments, the $GO_{x}$ -coated Janus displayed similar behavior [36,37].

Subsequently, the intermediate product I diffuses to the nearby layer composed of enzymes $E_{2}$ , where the second reaction produces B particles. At this point, $E_{1}$ and $E_{2}$ constitute an enzyme cascade reaction (ECR). The second stage of the reaction creates a gradient of B particles around the motor as well as the $E_{2}$ layer. Due to the different interactions of B particles with $E_{2}$ ( $\epsilon_{BE_{2}}<\epsilon_{\alpha E_{2}}$ ), the resulting asymmetrical gradient fields contribute to additional propulsion along the $\hat{u}$ direction, as shown in Figure 2a.

The influence of the area percentage of enzyme $E_{2}$ ( ϕ ) occupying the second layer on the self-propulsion velocity is investigated. At low ϕ values, $V_{u}$ increases slowly with increasing ϕ ; however, as ϕ approaches 0.5, $V_{u}$ grows rapidly. As shown in Figure 2a, $V_{u}$ reaches 0.0069 at ϕ=0.8 , indicating an increase of nearly 2.4 times compared to motors without cascade enzyme $E_{2}$ ( $V_{u0}=0.0029$ ). Figure 2b further demonstrates that at ϕ=1.0 , $V_{u}$ is approximately five times greater than that with ϕ=0 . This shows a significant enhancement in the enzymatic cascade efficiency as more enzyme $E_{2}$ is introduced.

This phenomenon can be rationalized as follows: Initially, the increase in motor velocity is modest because, as ϕ increases, the overall mass of the motor also increases, which in turn raises the drag force on the motor. Furthermore, when ϕ is small, the number of B particles produced is low, and the intermediate I particles diffuse away without being blocked by the second layer. Consequently, a weaker concentration gradient around the motor results in slower velocity growth. As ϕ increases, the concentration of I particles near enzyme $E_{1}$ becomes more confined by the second layer of $E_{2}$ , which prevents their diffusing away and gives rise to a stronger concentration gradient. This increases the efficiency of the enzymatic cascade, leading to higher propulsion arising from the self-diffusiophoretic mechanism.

The value of $R_{2}$ also affects the self-propelled velocity. In Figure 2b, we compare the effects of placing enzyme $E_{2}$ at the following three different positions: $R_{2}=3.95,\;4.2,$ and 4.35 . The results indicate that, at these different radii, $V_{u}$ varies with ϕ at the same scaling rate, following the power law, $V_{u}∼\phi^{4}$ . Notably, for the same ϕ , a smaller $R_{2}$ results in a faster $V_{u}$ . Through curve fitting, we obtain the following relationship:

(1) $$V_{u}=A\left(R_{2}\right)\phi^{4}+V_{u0},$$

where $A\left(R_{2}\right)=a-bR_{2}$ , with $a=5.05\times 10^{-2}$ and $b=0.98\times 10^{-2}$ . This relationship shows that as ϕ increases, $V_{u}$ rises more rapidly, highlighting the essential role of ECRs within this motor structure. Additionally, we found that as $R_{2}$ decreases, the $E_{2}$ layer is closer to the hemisphere coated by $E_{1}$ , facilitating the occurrence of an ECR. A similar phenomenon has been observed in previous experiments, where decreasing the distance between enzyme pairs ( $GO_{x}$ and HRP ) on DNA nanostructures resulted in enhanced activity [38,39].

**Figure 3:**
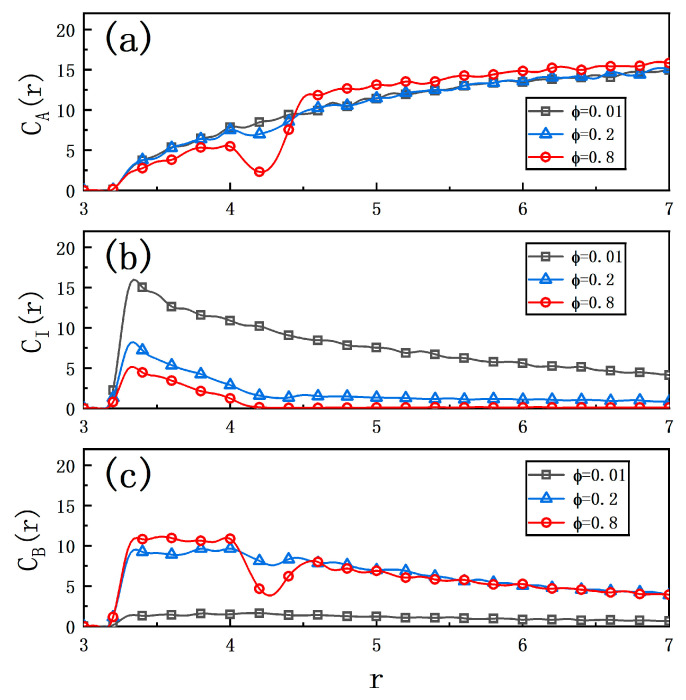
Radial concentration profiles of solvent particles: (**a**) A , (**b**) I , and (**c**) B around the catalytic hemisphere, represented as $C_{\alpha }\left(r\right)$ ( α=A, I, B ). The distributions are shown for different proportions of $E_{2}$ : ϕ=0.01 (squares), ϕ=0.2 (triangles), and ϕ=0.8 (diamonds). The radius $R_{2}=4.2$ is maintained across all measurements, which are based on 20 independent realizations.

In addition to enhancing self-propulsion, minimizing the toxic effects of intermediate products is a crucial factor in ECRs. This is particularly relevant in the widely studied $GO_{x}$ and Cat systems, where hydrogen peroxide ( $H_{2}O_{2}$ ) poses serious toxicity to cells, making it necessary to reduce its concentration in the solution. To provide insight into the motion mechanism and toxic effects arising from the asymmetric product distribution in this configuration, we perform a detailed analysis of the molecular concentration fields surrounding the motor. Figure 3 presents the radial distribution of the concentration field around the Janus motor. In Figure 3a, when ϕ is low ( ϕ=0.1 ), the concentration of particle A decreases near the motor due to the conversion reaction A →I . Consequently, a strong concentration gradient for particle I is observed in Figure 3b, which generates the propulsion force for the motor via this first enzymatic reaction. In this case, particles I can diffuse over considerable distances far from the motor. However, as a consequence, on one hand, the intermediate products are not effectively utilized, while on the other hand, their escape into the surrounding environment may result in toxic effects. As ϕ increases, the second enzymatic reaction becomes significant. In Figure 3a, we observe a growing concentration difference in particle A on both sides of enzyme $E_{2}$ . It is evident that at ϕ=0.8 , the dip in the concentration curve for particle A becomes pronounced. In the space between enzymes $E_{1}$ and $E_{2}$ , the concentration of I decreases quickly due to the reaction I →B as ϕ increases. Correspondingly, the concentration of particle B rises sharply, providing a robust propulsion force for the second reaction. This force stems from the concentration gradient of particle B around $E_{2}$ , as illustrated in Figure 3c, where the concentration difference is more evident at both ends of the dip. Furthermore, particle I not only fuels the second enzymatic reaction but also experiences a rapid decline in concentration outside enzyme $E_{2}$ ( $r>R_{2}+\sigma_{2}$ ) as ϕ increases, as seen in Figure 3b. When ϕ=0.8 , the concentration $C_{B}\left(r\right)$ approaches zero, indicating that particle I is effectively utilized and thus preventing the escape of toxic intermediate products.

**Figure 4:**
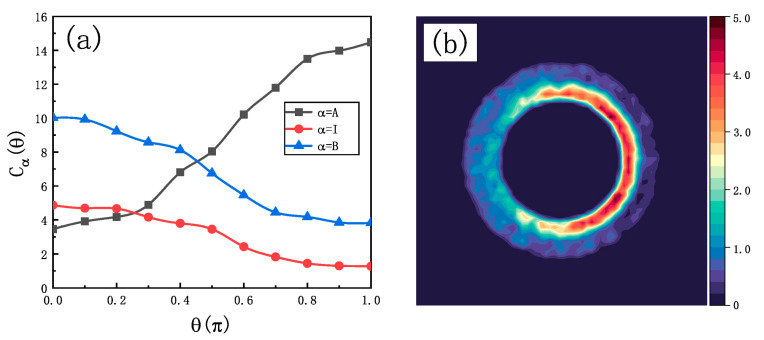
(**a**) Average angular concentration $C_{\alpha }\left(\theta \right)$ of A (squares), I (circles), and B (triangles), when ϕ=0.6 . θ is defined as the angle between the vector from the Janus center to the particle position and the vector $-\hat{u}$ . (**b**) Concentration distribution of I particles according to the present parameters. Data are averages of 40 independent realizations.

Aside from the radial distribution, the angular concentration distribution of the solvent particles around the motor provides an additional understanding of the propulsion mechanism and the origin of the increased velocity. Next, we analyze the angular concentration distribution $C_{\alpha }\left(r\right)$ ( α=A, I, B ) within a spherical shell with a radius range of 3.2<r<3.5 . This angular concentration of reactants and products allows for a more intuitive observation of the asymmetric concentration gradient surrounding the motor. As shown in Figure 4a, the $C_{\alpha }$ profiles are typical of Janus colloids where the largest gradient is in the vicinity of the equator ( $\theta =\frac{\pi }{2}$ ), where the two hemispheres meet. The concentration of particle A increases monotonically with the angle θ , reaching its maximum at θ=π . In contrast, the concentrations of particles I and B decrease on the opposite side of the Janus structure. This asymmetry in the angular distribution of those species at both ends of the Janus generates the propulsion force for the first enzymatic reaction, providing a clear mechanism for self-diffusiophoresis. The presence of the second enzyme confines the intermediate product I and the final product B within the space between enzymes $E_{1}$ and $E_{2}$ , thereby amplifying this asymmetry and correspondingly increasing the first propulsion force. Figure 4b clearly illustrates this phenomenon. There, we can observe that particles I are predominantly concentrated in the confined space, where they are well contained, with only a small portion gradually diffusing toward the inert hemisphere. Only a small amount of particle I is able to escape out of $E_{2}$ . Similarly, the asymmetric distribution around $E_{2}$ contributes to the second propulsion, while the confined environment further facilitates the enhancement of this second power.

Combining the insights from Figure 3 and Figure 4, we observe that under the self-diffusiophoresis mechanism, strong concentration gradients arise at both sides of the Janus sphere as well as that of the $E_{2}$ enzyme layer. This enhancement in the concentration gradients enables the motor to increase its velocity effectively by adding the $E_{2}$ enzyme layer. Furthermore, the presence of the $E_{2}$ enzyme layer prevents the leakage of intermediate product I particles, greatly enhancing the motor’s safety and environmental compatibility. These results not only contribute to a deeper understanding of the dynamic properties of colloidal motors but also provide new directions for developing efficient and safe enzyme-powered nanomotors. Detailed analysis of the radial and angular concentration distributions at different ratios ϕ finds that as the proportion of $E_{2}$ increases, the concentration gradients of A and B particles around the motor are significantly enhanced, while the concentration of I particles is markedly decreased. These changes indicate that the presence of $E_{2}$ not only boosts the propulsion efficiency of the motor but also enhances overall system performance by reducing the generation of harmful intermediate products. Additionally, numerical simulations allow us to clarify phenomena that are challenging to assess experimentally, such as the influence of concentration gradients on propulsion velocity and the distribution of I particles under different configurations.

**Figure 5:**
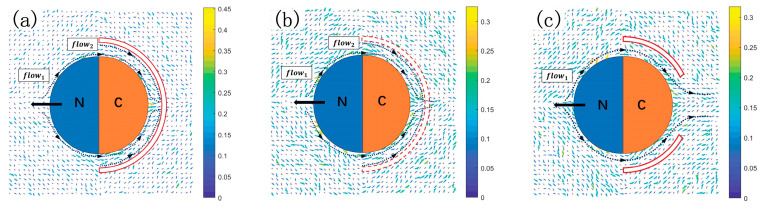
Velocity flow field distribution of solvent particles around the Janus sphere. The arrows indicate the movement direction, while the dashed lines represent the flow fields of the surrounding fluid. The N hemisphere contains enzyme $E_{0}$ , and the C hemisphere contains enzyme $E_{1}$ . Flow field distributions are shown for different ϕ values, ϕ=1.0 (**a**) and ϕ=0.8 (**b**), where $E_{2}$ is uniformly distributed. The region within the red line indicates the zone where enzyme $E_{2}$ is located at $R_{2}=4.2$ . Panel (**c**) illustrates the flow field distribution of solvent particles at ϕ=0.8 after removing a portion of $E_{2}$ from (**a**) to create an opening near θ=0 . Data are averages of 40 independent realizations.

This ECR motor configuration contributes to the utilization of fuel A particles. We perform a statistical analysis of the velocity field around the Janus sphere. Figure 5a,b illustrate the flow field distributions for ϕ=1.0 and ϕ=0.8 , respectively. The flow fields induced by the moving motor configuration are clearly shown in these two figures. The flow field surrounding the sphere, labeled as $flow_{1}$ , is generated by the movement of the Janus colloid. Additionally, a new flow field, $flow_{2}$ , is induced near the inner surface of $E_{2}$ layer. The flow field, resulting from the combined effects of the open hemispherical structure and the motor’s self-propulsion, facilitates the influx of fuel particles A into the regions between $E_{1}$ and $E_{2}$ , enhancing the self-power capability. This structure confines the flow field of intermediate product I , optimizing its utilization to enhance propulsion. The influence of this flow field is enhanced as ϕ increases. We illustrate this point by comparing the flow fields around the motor at different ϕ in Figure 5a,b. When ϕ=1.0 , there is a clear influx of A particles, while the I and B particles are confined. As ϕ decreases, as shown in Figure 5b for ϕ=0.8 , $flow_{1}$ and $flow_{2}$ converge near θ=0 and subsequently flow out through the gap among $E_{2}$ beads (as indicated in the corresponding flow fields). This convergence leads to a reduction in the asymmetric gradient and, consequently, a decrease in velocity. Therefore, a larger ϕ effectively confines the fuel and intermediate products, significantly increasing the propulsion. The flow field effect provides a qualitative basis for the rapid increase in $V_{u}$ with ϕ , indicating a pronounced dependence on ϕ (Figure 5b shows $V_{u}∼\phi^{4}$ ). The influence of different motor structures on propulsion is analyzed by comparing flow field distributions at a same ϕ . In Figure 5c, we remove some $E_{2}$ near θ=0 from Figure 5a to achieve ϕ=0.8 . The flow field near θ=0 clearly shows solvent particles flowing out the gap, a phenomenon that may significantly reduce the motor’s self-propulsion. Numerical simulations confirmed the following point: The self-propulsion velocity decreases from $V_{u}=0.08$ in Figure 5b to $V_{u}=0.066$ in Figure 5c.

## 3. Materials and Methods

To set up the model, we establish a Janus sphere with radius R , where $N_{in}$ particles, each with mass $m_{in}$ , are randomly distributed throughout the sphere interior. Additionally, the $N_{out}$ enzyme beads, each with mass $m_{out}$ and radius $\sigma_{1}$ , are uniformly positioned on its surface. The particles and beads are all interconnected by spring-like forces, which can be calculated with the equation:

(2) $$U\left(r_{ij}\right)=\frac{1}{2}k_{s}{\left(r_{ij}-r_{ij}^{0}\right)}^{2},$$

where $r_{ij}$ represents the distance between particles (or beads) i and j , $k_{s}$ denotes the spring stiffness coefficient, and $r_{ij}^{0}$ is the initial equilibrium separation distance. The total mass of the motor is $M=N_{in}\times m_{in}+N_{out}\times m_{out}$ , where $m_{in}$ and $m_{out}$ are chosen to satisfy the expressions $M=\frac{4}{3}n_{s}\pi R^{3}$ to ensure that the motor is approximately neutrally buoyant, and $I=\frac{2}{5}MR^{2}$ , respectively, where I is the moment of inertia, and $n_{s}$ is the solvent particle number density in the solution. Numerical simulations indicate that the positional fluctuations of the enzyme beads during motion are minimal, ensuring that the motor sphere preserves its spherical geometry.

One hemisphere is covered with inert enzyme beads, labeled as $E_{0}$ , with a total count of $N_{E_{0}}$ . In contrast, the other hemisphere is coated with catalytically active enzyme beads ( $E_{1}$ ) and contains $N_{E_{1}}$ beads. Then, a Janus collide model is set up. Furthermore, an outer shell is constructed on the outer layer, onto which a different type of enzyme ( $E_{2}$ with a number of $N_{E_{2}}$ ) is immobilized, as illustrated in Figure 1. It is well known that the self-propulsion capability is influenced by the number of enzyme modifications on the surface [26,40,41]. Notably, when portions of the Janus surface are assigned to a second type of enzyme to form a cascade reaction, the total surface area of the colloid remains unchanged, consequently limiting the total enzyme number. Our layered design provides additional spatial capacity, enabling an increased enzyme density on the surface. This model can be experimentally fabricated by sequentially layering multiple enzymes on the surface, such as $GO_{x}$ and Cat . As demonstrated by Pan et al., a platinum ( Pt ) layer is initially deposited on the Janus colloid, followed by an outer modification with $GO_{x}$ to promote a cascade reaction [42].

The motor is placed within a cubic system with volume $V=L_{x}\times L_{y}\times L_{z}$ , which is filled with a large number of point-like solvent particles, typically $10^{6}$ , each with mass $m_{s}$ . Initially, all the solvent particles are designated as fuel particles ( A ), randomly distributed outside the motor, with velocities plotted from the Maxwell–Boltzmann distribution and characterized by system temperature T . Upon collision between fuel particles A and the catalytically active enzyme $E_{1}$ , an irreversible reaction occurs, as follows:

(3) $$A+E_{1}\to I+E_{1},$$

where I denotes the intermediate product. Subsequently, when the I particles collide with enzyme $E_{2}$ , a secondary reaction is triggered, as follows:

(4) $$I+E_{2}\to B+E_{2},$$

where B denotes the final product. These two reactions constitute a cascade between enzymes $E_{1}$ and $E_{2}$ on the motor surface. To mimic a sustained fuel supply, the product particles I and B are regenerated into A through the following reactions:

(5) $$B\overset{k_{b}^{B}}{\to }A\;\;\;and\;\;I\;\overset{k_{b}^{I}}{\to }\;A,$$

where $k_{b}^{B}$ and $k_{b}^{I}$ represent the reaction rate constants. Experimentally, continuous fuel supply can also be achieved by feeding fresh fuel into the system and removing reaction products as required [2,4,15]. This cascade model is analogous to the well-studied glucose oxidase–catalase ( GOx-Cat ) system, where glucose ( Glc ) produces hydrogen peroxide ( $H_{2}O_{2}$ ) in the presence of glucose oxidase ( GOx ), while the resulting $H_{2}O_{2}$ is then decomposed by catalase ( Cat ) to yield oxygen ( $O_{2}$ ). Both stages contribute to motor propulsion and achieve high conversion efficiency [27,30,43].

The evolution of the system is simulated by a hybrid molecular dynamics–multi-particle collision dynamics (MD–MPC) approach, as described in [44]. Interactions between the solvent particles and the motor are governed by a repulsive 6–12 Lennard–Jones potential, as follows:

(6) $$V_{\alpha \beta }\left(r\right)=4\epsilon_{\alpha \beta }\left[{\left(\frac{\sigma }{r}\right)}^{12}-{\left(\frac{\sigma }{r}\right)}^{6}+\frac{1}{4}\right]\Theta \left(r_{c}-r\right),$$

where $\Theta \left(x\right)$ denotes the Heaviside step function, and $r_{c}=2^{1/6}$ defines the cutoff radius. The term $V_{\alpha \beta }$ represents the interaction potential between the different types of solvent particles α ( α=A, I, B ) and the motor surface enzymes β ( $\beta =E_{0},E_{1},E_{2}$ ). The interaction parameters are set such that $\epsilon_{I\beta }=\epsilon_{BE_{0}}=\epsilon_{BE_{1}}=\epsilon_{AE_{2}}>\epsilon_{AE_{0}}=\epsilon_{AE_{1}}>\epsilon_{BE_{2}}$ , to simulate the self-propulsion of the motor based on self-diffusiophoresis.

In molecular dynamics (MD) with a time interval $\tau_{MD}$ , particle motion is executed by the velocity Verlet algorithm, which numerically integrates Newton’s equations of motion. The ECRs are considered at each MD time. A multiparticle collision (MPC) dynamics step is performed after 100 MD steps. During the MPC step, solvent particle interactions are governed by collision [45,46]. They are assigned to a lattice with cell size $a_{0}$ . Independent multi-particle collision processes then occur in each cell, where solvent particles within a given cell undergo velocity changes due to the random rotational collision rule. The post-collision velocity for the i -th particle in cell ξ is updated as follows [45,46]:

(7) $$V_{i}\left(t+t_{MPC}\right)=V_{cm}\left(t\right)+{\hat{\omega }}_{\xi }\left(\theta \right)\left(v_{i}\left(t\right)-v_{cm}\left(t\right)\right),$$

where ${\hat{\omega }}_{\xi }\left(\theta \right)$ represents the rotation matrix, and $V_{cm}\left(t\right)$ is the center-of-mass velocity of cell ξ . Periodic boundary conditions are applied along the x , y , and z directions. The system evolves iteratively through alternating MD and MPC steps.

MPC–MD dynamics is microcanonical and preserves the basic conservation laws, such as conserving mass, momentum, and energy, as well as phase space volumes. Therefore, hydrodynamic interactions are properly taken into account and no additional assumptions about friction coefficients or random forces need to be made. Thermal fluctuations in low Reynolds number environments are considered when simulating dynamics of nanomotor [45].

In our simulations, all quantities are reported in dimensionless units based on the energy ε , mass $m_{s}$ , and distance $a_{0}$ parameters, as follows: $t{\left(m_{s}a_{0}^{2}/\varepsilon \right)}^{1/2}\to t$ , $r/a_{0}\to r$ , and $k_{B}T/\varepsilon \to T$ . The system temperature T is set to $\frac{1}{6}$ . The diffusion coefficient of the substrate molecules D=0.0899  according to the relation $D=\frac{k_{B}Tt_{MPC}}{2m_{s}}\left(\frac{3n_{s}}{\left(n_{s}-1+e^{-n_{s}}\right)\left(1-cos\varphi \right)}-1\right)$ [45]. The initial density of solvent particles is $n_{s}=20$ . A rotation angle of $\varphi =\frac{\pi }{2}$ and a collision time interval of $t_{MPC}=0.5$ are employed. Additional simulation parameters are as follows: $L_{x}=L_{y}=L_{z}=30$ ; $N_{in}=2681$ ; $m_{in}=0.44$ ; $m_{out}=1.50$ ; $m_{2}=0.64$ ; $\epsilon_{AE_{0}}=\epsilon_{AE_{1}}=0.2$ ; $\epsilon_{I\beta }=\epsilon_{BE_{0}}=\epsilon_{BE_{1}}=\epsilon_{AE_{2}}=5.0$ ; $\epsilon_{BE_{2}}=0.1$ ; $t_{MD}=0.005$ ; spring constant $k_{s}=60$ ; and fluid rate constants $k_{b}^{B}=k_{b}^{I}=0.001$ . To ensure Galilean invariance, grid shifting is applied within the MPC method.

## 4. Conclusions

Strong propulsion and safety are central concerns in the design of self-propelled micro- and nanomotors. ECR-powered small motors are considered as a viable solution to achieve these goals. We propose a motor configuration that exhibits both strong power and enhanced safety features. By modifying Janus spheres with two layers of enzymes to facilitate ECRs, this configuration presents interesting advantages. On one hand, this configuration, distinct from the traditional enzyme modification methods, increases the available surface area for enzyme attachment, thereby enhancing the propulsion force. Through the cooperative effects of secondary enzymes, the propulsion of both the primary and secondary enzymes are enhanced, thereby effectively increasing the overall force. Furthermore, this structure efficiently prevents the escape of intermediate products, significantly improving safety.

Simulations reveal that the velocity increases rapidly with the density of enzyme $E_{2}$ , following a relationship of $V_{u}∼\phi^{4}$ . Through analyzing the radial and angular concentration fields around the motor, the study sheds light on the underlying mechanisms behind the enhanced propulsion and safety. Additionally, the contribution of the fluid field to the velocity is explored. Our design provides a basic model for further studies and experiments.

Small motors powered by ECRs have been applied in various potential fields, demonstrating multiple functions. Among these, motion-based diagnostics and therapeutics are an important direction. For example, $GO_{x}$ and HRP enzymes can detect dynamic changes in intracellular glucose concentration at low levels [28], while diamine oxidase ( DAO ) and Cat enzymes can be used for the immune therapy of diseases targeting histamine [47]. Our results capture the main kinetics of ECRs motor. Furthermore, this novel configuration can contribute to the design of ECR-powered motors with more efficient propulsion and safer biocompatibility.

## Data Availability

Data is contained within the article.

## Abbreviations

The following abbreviations are used in this manuscript:

ECRs	enzyme cascade reactions
MD	molecular dynamics
MPC	multiparticle collision dynamics
GOx	glucose oxidase
Cat	catalase
HRP	horseradish peroxidase
BOD	bilirubin oxidase
Cyt c	cytochrome c
DAO	diamine oxidase
