# Correction: Hu et al. Dynamics of Nanomotors Propelled by Enzyme Cascade Reactions. *Int. J. Mol. Sci.* 2024, *25*, 12586

**DOI:** 10.3390/ijms26020603

**Published:** 2025-01-13

**Authors:** Jia-Qi Hu, Rui Zhao, Ru-Fei Cui, Jian-Long Kou, Jiang-Xing Chen

**Affiliations:** 1 School of Physics, Hangzhou Normal University, Hangzhou 311121, China; 2 Department of Physics, Hangzhou Dianzi University, Hangzhou 310027, China; 3 Institute of Condensed Matter Physics, Zhejiang Institute of Photoelectronics and Zhejiang Institute for Advanced Light Source, Zhejiang Normal University, Jinhua 321004, China; kjl@zjnu.cn

There was an error in the original publication [1]. A paragraph was missing from the text.

A correction has been made to **2. Results and Discussion. Paragraph Number 1**: a new paragraph has been inserted before the first paragraph of this section.

Our investigations of motor dynamics propelled by ECRs use a coarse-grain microscopic description of the entire system. We consider a colloid with two types of enzymes coated on its surface. One of the enzymes $E_{0}$ is inert, while the other, enzyme $E_{1}$ (mimicking $GO_{x}$), catalyzes the fuel A (mimicking glucose) into an intermediate product I (mimicking $H_{2}O_{2}$). Such Janus colloid has been fabricated in recent experiments. In addition, we construct a special structure by grafting another enzyme, $E_{2}$ (mimicking Cat), onto the outer layer of $E_{1}$, which catalyzes the intermediate product I into the final product B (mimicking $O_{2}$). A schematic diagram of the motor configuration is presented in Figure 1. The nanomotor is operated by a diffusiophoretic mechanism where the ECRs on the active enzymes produce concentration gradients that drive propulsion. The solution in which the motor moves contains fuel A as well as products I and B particles. These species interact with the motor through intermolecular potentials and determine how the motors move in the presence of chemical gradients produced by the motors themselves. Additional information concerning the motor configuration and the simulation is given in the Section 3.

The authors state that the scientific conclusions are unaffected. This correction was approved by the Academic Editor. The original publication has also been updated.
